# Phylogenetic, morphological and niche differentiation unveil new species limits for the big brown bat (*Eptesicus fuscus*)

**DOI:** 10.1098/rsos.231384

**Published:** 2024-02-07

**Authors:** Pedro Ivo Mônico, J. Angel Soto-Centeno

**Affiliations:** ^1^ Department of Earth and Environmental Sciences, Rutgers University, Newark, NJ 07102, USA; ^2^ Department of Mammalogy, American Museum of Natural History, New York, NY 10024, USA

**Keywords:** Caribbean, Chiroptera, *Eptesicus dutertreus*, insular, species delimitation, Vespertilionidae

## Abstract

Phylogeographic accounts of mammals across fragmented landscapes show high levels of genetic, morphological and ecological variation. The big brown bat (*Eptesicus fuscus*) widely spans mainland landmasses from Canada to Ecuador and Colombia, and the insular Caribbean through The Bahamas and Greater and Lesser Antilles. Given the distribution of *E. fuscus*, we hypothesized that insular lineages could represent a different species aided by isolation. We assessed species limits by capitalizing on available mitochondrial and genomic data. Novel morphological and spatial datasets were produced to examine limits phenotypically and whether ecological niches could be associated with differences between groups. Phylogenetics strongly supported the Caribbean as unique compared to the mainland. Genomic data indicated high levels of genetic structure within the Caribbean and no detectable admixture of the Caribbean with continental lineages. Similarly, the Caribbean group shows high phenotypic disparity, and niche models revealed differences in habitat suitability between groups, concordant with the phylogenetic results. This study uncovered signals of divergence supporting the Caribbean clade of *E. fuscus* as unique through an integrative framework. We endorse re-evaluating the taxonomic status of Caribbean big brown bats as *Eptesicus dutertreus*. This recognition can help promote local conservation plans for insular lineages of big brown bats.

## 1. Introduction

Species delimitation plays a central role in systematics and taxonomy, and the products of this endeavour have broader implications for biodiversity science and conservation [1]. Advances in genetic and genomic data collection and analyses have led to more robust examinations of species limits under broad phylogeographic frameworks [2,3]. Notwithstanding, proper evaluation of species limits is often overlooked, and the process is challenged by a lack of knowledge of the taxonomic units examined, especially for taxa that show little morphological disparity and/or are considered cryptic [4]. Hidden diversity is perhaps greatest in species that occupy broad geographic ranges across fragmented landscapes because such taxa frequently occur over a range of environmental and geographic features that may influence genetic and morphological variation [5]. Documenting patterns of hidden diversity is imperative to provide thorough biogeographic and taxonomic accounts. As habitats and species become ever more threatened by anthropogenic factors, proper delimitation of species is critical to make evidence-based conservation and management decisions about biodiversity.

Before the broad availability of genomic methods, many studies either relied entirely on morphology (e.g. morphometrics or phenotypic trait comparisons) [6] or employed single-gene barcode methods to document hidden patterns of diversity and evaluate species limits [7,8]. More recently, the integration of multiple methods to evaluate taxonomic questions has become more prevalent. These approaches borrow from many technological advances, such as access to digitized museum collections and databases, refinement of fine-scale DNA sequencing procedures, and tools developed with geographical information systems [9–11]. Thorough phylogeographic evaluations using either multilocus or genomic datasets have flourished in recent years [12–15]. Still, taxonomic proposals and species limits sometimes are evaluated based on limited evidence approaches [16,17]. Relying solely on one approach could provide an incomplete perspective of taxonomic diversity [18,19]. Thus, it is essential to leverage multiple approaches and evaluate their congruence to simultaneously avoid unjustified taxonomic inflation or underestimating the history of biological lineages [20–22]. The combination of various independent sources of data (e.g. ecology, genetics and morphology) can provide valuable knowledge of the evolutionary trajectory of species and the drivers of speciation [22,23].

Phylogeographic accounts of mammals across insular fragmented landscapes support high levels of genetic variation and/or population structure of species-like lineages [24–28]. Compared to non-volant mammals, bats are an exemplary model for studying patterns of diversification and structure. Because of their capacity for powered flight, bats show high dispersal ability, and many species have overcome oceanic straits to occupy mainland and insular land masses. Some bats are renowned for their morphological diversity resulting from shifts in their ecological preferences, such as those in Neotropical leaf-nosed bats (family Phyllostomidae) [29]. Nonetheless, studies also suggest high levels of cryptic diversity among many species [13,26].

The big brown bat (*Eptesicus fuscus*) is a widely distributed species spanning mainland landmasses from Canada to Ecuador and Colombia and the insular Caribbean through The Bahamas and Greater and Lesser Antilles [30,31]. This species is common and readily identifiable throughout its range by its characteristic brown pelage and dark brown wing membranes. There are currently 13 recognized subspecies of *Eptesicus fuscus* [32]. Six subspecies occur across the mainland (*E. f. fuscus* Beauvois, 1796; *E. f. miradorensis* Allen, 1866; *E. f. peninsulae* Thomas, 1898; *E. f. bernardinus* Rhoads, 1902; *E. f. osceola* Rhoads, 1902; *E. f. pallidus* Young, 1908), and seven occur throughout the Caribbean islands (*E. f. dutertreus* Gervais, 1837; *E. f. bahamensis* Miller, 1897; *E. f. wetmorei* Jackson, 1916; *E. f. hispaniolae* Miller, 1918; *E. f. lynni* Shamel, 1945; *E. f. petersoni* Silva-Taboada, 1974). Some taxonomic decisions used herein should be justified. Recently, Ramírez-Chaves *et al*. [17] proposed to elevate *E. f. miradorensis* to species level based on genetic distance evidence in two individually analysed mitochondrial genes and some morphological characters. We do not follow this taxonomic change here and retain its subspecific designation as in Simmons & Cirranello [32]. Another species, *E. guadeloupensis* from the Caribbean, was described based on morphological characters of three specimens [33]. This species appears to be a rare bat on Guadeloupe Island [34]. A recent review including two specimens seems to confirm its uniqueness [35] but a contrasting hypothesis was presented by Yi & Latch [36]. In the absence of additional data to evaluate, we treat *E. guadeloupensis* as a valid taxon in this paper. Timm & Genoways [37] questioned the validity of the Jamaican endemic, *E. lynni*. Given that recent studies showed that this taxon is associated with *E. f. hispaniolae*, we followed the taxonomic recommendation of Simmons & Cirranello [32] to treat the Jamaican lineage as a subspecies of *E. fuscus*. Finally, in a different taxonomic study on the genus *Eptesicus*, the specific epithet ‘fuscus’ was restricted to mainland North and Central America and the Greater Antilles [35].

Phylogeographic accounts of *E. fuscus* revealed high levels of genetic diversity and complex population level relationships with considerable genetic distances between continental and insular forms [14,38]. Beyond that, no study has directly examined the potential for a species complex within *E. fuscus* under an integrative delimitation framework spanning the heterogeneity of the mainland and the Caribbean groups. We studied the genetic, morphological and ecological niche variation of the big brown bat (*Eptesicus fuscus*) with a focus on Caribbean populations. The insular Caribbean region shows high habitat and topographic heterogeneity and landmasses with varying levels of isolation, which promoted patterns of population divergence and speciation in many taxa (e.g. [39]). Given the wide distribution of *E. fuscus* across mainland and insular landscapes, we hypothesized that species diversity in this taxon may be underestimated. In this study, we used multiple lines of evidence to avoid the pitfalls of single-method taxonomic change proposals [22]. First, we assessed genetic species limits by capitalizing on the available single-gene data from Turmelle *et al*. [38] and genomic data from Yi & Latch [14]. We also produced a novel morphological dataset to examine species limits phenotypically under two machine learning methods. Finally, we used an ecological niche modelling and niche quantification approach to explore whether climatic factors could be associated with observed differences among insular and continental groups. We predicted that the Caribbean lineages constitute a geographically structured group distinguishable from the continental ones by genetic, morphological, and ecological characteristics.

## 2. Material and methods

### 2.1. Phylogenetic reconstruction and species delimitation

#### 2.1.1. Analysis of mitochondrial DNA

We used the mitochondrial dataset of the NADH dehydrogenase subunit 2 (ND2) gene from Turmelle *et al*. [38], obtained from NCBI GenBank (electronic supplementary material, table S1). Bayesian inference implemented in MrBayes 3.2.7a [40] was used to determine the phylogenetic relationships of mitochondrial lineages (electronic supplementary material, table S1) between continental and insular *E. fuscus* within the CIPRES (Cyberinfrastructure for Phylogenetic Research) Science Gateway v3.3 [41]. We selected the best nucleotide substitution model (GTR + I + G) using jModelTest and implemented two runs with four Markov chains for 1 × 10^7^ generations, sampling every one thousand generations. We assessed the convergence among Bayesian reconstructions by evaluating the average standard deviation of split frequencies (less than 0.01) and generated a 50% majority rule consensus tree to calculate the posterior probabilities. Furthermore, we estimated a maximum likelihood phylogenetic tree using IQ-TREE v2.0.3 [42] after appropriately selecting the best nucleotide substitution model (TIM + F + G4), using the model finder plus (-MPF) option [43].

We predicted that the Caribbean lineages of *E. fuscus* represent one or more species. To test this under the mitochondrial perspective, we evaluated their limits using the single locus coalescent approach, multi-rate Poisson tree processes (mPTP) [44]. We performed this analysis in the mPTP MCMC Web server (https://mcmc-mptp.h-its.org/mcmc/) using the ML phylogenetic tree. The Markov chain Monte Carlo (MCMC) runs were sampled every 1000 generations (10% burn-in) for 5 **×** 10^6^ generations. We performed three analyses with distinct starting delimitation models: null model (considering all lineages as constituting one species), maximum likelihood model (MLE-based delimitation), and random model (arbitrary delimitation). We included the intra-specific differences among coalescence rates with a minimum branch length of 0.0001 by using the option -multi in every analysis.

#### 2.1.2. Analysis of restriction-site-associated DNA sequences

Available genomic data were obtained from a published phylogeographic account on *E. fuscus* [14] to go beyond the single-gene mitochondrial approach and provide a backbone of species delimitation for the Caribbean (electronic supplementary material, table S2). These data were produced using a bestRAD protocol for developing single nucleotide polymorphisms (SNPs) per individual, and libraries were sequenced in Illumina NovaSeq 6000 (for sequencing details and SNP assembly, see [14]). Specifically, our analysis focused on a reduced dataset of 4076 SNPs for 27 individuals from the Caribbean, Mesoamerica, and southeastern United States, including a single individual of *Neoeptesicus furinalis* (*Eptesicus furinalis* op. cit. [14] from Bolivia as an outgroup, Efur01). The data contained no sequence gaps, were filtered for three minimum allele counts, and the vcf file was obtained from the Dryad repository (doi:10.5061/dryad.xsj3tx9h3). Finally, we used the Python script ‘vcf2phylip 2.0’ [45] to produce fasta and nexus formatted alignments for phylogenetic and species delimitation analyses. A maximum likelihood phylogeny was estimated using IQ-TREE v2.0.3. We used the model finder plus (-MPF) option [43] within IQ-TREE v2.0.3 to obtain the best model of nucleotide substitution in a preliminary run, which resulted in (HKY + F + I). This model was then implemented in a full tree search, including 1000 ultrafast bootstrap replicates with 1000 SH-like aLRT (approximate likelihood ratio test) to assess branch support [46]. The resulting phylogeny was rooted manually using a single individual from *N. furinalis* and plotted in FigTree 1.4 (http://tree.bio.ed.ac.uk/software/figtree/).

As a second step, we performed a discriminant analysis of principal components (DAPC) to test for genetic clusters and population structure among groups. This method is helpful in identifying differences between groups while minimizing variation within each group [47]. To ensure that all variables submitted to DAPC were uncorrelated, we performed a prior principal components analysis (PCA) using the same 27 individual *E. fuscus* SNP dataset [47]. We performed the DAPC group differentiation estimates using the adegenet v2.1.10 R package [48], and created structure plots, population assignments and posterior membership probabilities for the analysed partitions of *E. fuscus*.

To evaluate the structure of the phylogenetic hybridization networks between continental and insular taxa, we used SplitsTree v4.19 [49]. This framework aims to evaluate whether there are signals of a phylogenetic network or reticulation among related groups. These analyses compute a reticulate network that explains the molecular sequence evolution using minimal reticulations [49]. The products are connected splits or isolated branch patterns, which can be tested under the hypothesis of divergence of compatibility between groups. We used the same SNP dataset, manually rooted using *N. furinalis*, to produce a phylogenetic network of branched trees.

Finally, we constructed a coalescent species tree from the multilocus unlinked SNP data using SVDquartets [50], implemented in PAUP* [51]. This method assumes that each site in the alignment has its underlying gene tree generated under the coalescent model from the species tree [52]. A similar analysis using the 4079 SNP loci data was performed by [14]. Herein, we specifically focused it on the species delimitation of Caribbean lineages. We assigned the geographical partitions as the taxonomic units and evaluated all 448 quartets with 100 bootstraps to build a 50% majority-rule consensus tree. The tree was manually rooted using *N. furinalis* as an outgroup and visualized in FigTree 1.4.

### 2.2. Phenotypic species delimitation from morphological data

We assessed the morphological variation patterns associated with geography in *E. fuscus*, specifically predicting that the Caribbean group is a diagnosable species. We measured 16 craniodental characters [53] from 88 specimens deposited in the American Museum of Natural History (AMNH) and Florida Museum of Natural History (FLMNH) mammalogy collections. These specimens encompassed insular populations in The Bahamas, Cuba, Hispaniola and Puerto Rico, and continental populations of Florida and Louisiana (USA), Mexico, Guatemala, Honduras, Colombia and Venezuela (electronic supplementary material, table S3). All measurements were taken with digital callipers (Mitutoyo, Japan) and rounded to the nearest 0.01 mm. Morphological groups were assigned following three geographic regions and the specimens examined included representative individuals from the Caribbean (*N* = 52), Mesoamerica (*N* = 24) and southeastern United States (*N* = 12).

We were unable to measure some characters of a few partially damaged specimens. Thus, we used the R package mice v3.15.0 [54] to perform a multivariate imputation by chained equations. To reduce bias, we partitioned the data by geographic group and ensured that missing data did not exceed 30% of the total of each partition [55]. This technique allowed us to fill in any missing data while maintaining the original relationships among traits [55]. Furthermore, this helped to maximize the sample size and extract as much morphometric information as possible from each group. Data were normalized using a log-transformation.

To isolate the possible effect of differences between sex (i.e. sexual dimorphism), we first conducted a PCA comparing male versus female *E. fuscus* in our dataset (*N* = 77). We then examined the levels of phenotypic distinctiveness among three geographical partitions of *E. fuscus*, testing the hypothesis of distinguishable groups based on morphological measurements. We used a supervised machine learning algorithm to perform a linear discriminant analysis (LDA) in the R package ‘MASS’ v.7.3.54 [56]. These LDA classification models were trained using a random 75/25% training/testing partition of the total dataset, followed by a k-fold cross-validation approach of 5 replicates. A confusion matrix was generated to estimate LDA model accuracy (i.e. how individuals were assigned to the geographical groups), which was then statistically compared to the no-information rate [57]. We scaled and centred the data, then plotted the first two linear discriminants (LD1 and LD2) in a two-dimensional plot to visualize the species' phenotypic limits and evaluate the variability among the three geographical groups of *E. fuscus*. A separate PCA was performed to confirm the phenotypic groups under an unsupervised machine learning method. We also performed 100 bootstrap replicates on the PCA using the R package bootSVD to compute 95% confidence intervals (CI) as a measure of stability of the PCA results. Finally, measures of central tendency were used to explore phenotypic variation in maxillary tooth row, dentary length, and rostral length, which contributed the most to the separation of each geographic group (see §3.2).

### 2.3. Environmental differences across geography

We predicted that environment could play a role promoting diversification. We developed a hypothesis testing framework to examine if there would be a difference in suitable habitats between the Caribbean and continental groups. For this, we collected georeferenced occurrence points of *E. fuscus* from the online database Global Biodiversity Information Facility (www.gbif.org) and obtained additional georeferenced occurrences from published literature [58–61]. We refined this dataset by removing non-georeferenced and duplicated records from the final analyses. We then performed spatial filtering to reduce potential geographical bias and auto-correlation using the spThin R package v 0.2.0 [62]. Locality records were plotted in QGIS 3.26 to correct georeferencing errors, and the final dataset for modelling contained 404 observations (electronic supplementary material, figure S1).

We followed the three geographic grouping described above to designate biologically relevant data partitions. This approach has been shown to increase performance when modelling multiple subspecies or species complexes [63]. The partitions included lineages from the Caribbean (represented by five subspecies: *E. f. bahamensis* (The Bahamas), *E. f. dutertreus* (Cuba), *E. f. hispaniolae* (Hispaniola), *E. f. lynni* (Jamaica), and *E. f. wetmorei* (Puerto Rico)), Mesoamerica (*E. f. miradorensis*), and southeastern United States (*E. f. osceola*).

Presence-only data were used to estimate the relationship between species occurrences and their associated environmental conditions, exploring potential ecological differences [64]. We generated ecological niche models (ENMs) using Maxent v3.4.4 in the R package ‘dismo’ v1.3–5 [65]. We used the ‘ENMeval’ v2.0.1 package to select the best model parameters [66,67]. The ‘ENMeval’ package provides multiple data partitioning tools, which allow building models with different algorithm settings and evaluating their performance. We explored multiple combinations of feature classes (FC = linear, quadratic, hinge, product, threshold) and regularization multipliers (i.e. beta multiplier or RM) ranging from 1 to 3. We selected the best set of parameters (FC and RM) to fit the data to models based on the corrected Akaike's information criterion value (AICc).

We used present-day bioclimatic data ‘WorldClim1’ in 2.5 arc minute resolution (about 5 km; http://www.worldclim.org/) [68]. One key aspect of correctly estimating niches is collecting the appropriate extent of background areas. Generally, assembling the appropriate bioclimatic conditions improves model accuracy [69]. First, we selected the background localities by creating a customized buffered polygon around the species' occurrences of each group partition and extracting the associated climate information. Then, we used the ‘raster’ R package [70] to extract the climate data from 10 000 random background points for each partition.

Two indices were used to assess model performance: AUC and Boyce index [71–73]. First, we evaluated each partition's model performance using the area under the receiving operating characteristic curve (AUC). This index ranges from 0 to 1, where values close to 1 represent excellent performance, values ≤ 0.5 are considered no better than random predictions, and models with values > 0.7 are typically considered of good performance [74]. Using only the AUC value has been deemed unreliable for estimating the performance of presence-background models [75]. Therefore, we also used the Boyce index to evaluate model robustness and deviation from randomness [72,76]. This index ranges from −1 to 1, with positive values near one indicating that predictions are consistent with the distribution of presences in the evaluation dataset. All final model configurations and summary statistics can be found in table 1.

**Table 1.** Occurrence points, feature classes, regularization multiplier, AUC values, and Boyce index from the ecological niche models produced in Maxent V3.4.4. for the three geographical partitions of *Eptesicus fuscus*. Feature classes indicate the different types of curves fitted by the Maxent model. These include linear (L), quadratic (Q), hinge (H), and product (P).

geographical partition	occurrence points	feature classes	*β* multiplier	AUC	Boyce
Caribbean	164	LQHP	1	0.738	0.997
Mesoamerica	219	LQ	1	0.819	0.985
southeastern US	21	LQHP	3	0.810	0.914

To test the hypothesis that environmental factors may help explain the divergence observed among groups, we performed niche similarity tests using the ‘ENMTools’ R package [77]. We performed a niche equivalency test to evaluate whether the niches are distinct or compatible among the Caribbean and mainland groups. Additionally, we performed a symmetrical background test to identify if the differences in environmental distributions reflect divergence in ecological niche tolerances or preferences. Our analysis compared Schoener's *D* overlap values to a null distribution based on 100 replicates for each test [78]. Running both tests helps quantify the environmental conditions where the species occurs while assembling surrounding areas. To determine whether niches were equivalent, we examined if the Schoener's *D* overlap was significantly lower than overlap values in the null distribution.

## 3. Results

### 3.1. Molecular phylogenetic and species limits analyses

**Figure 1.**
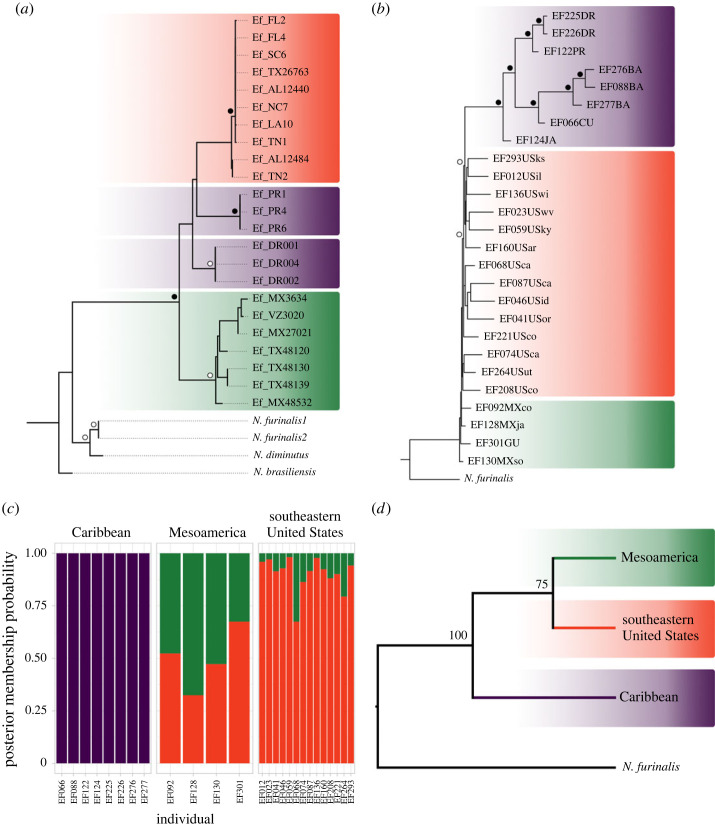
(*a*) Mitochondrial and (*b*) genomic SNPs IQTree maximum likelihood phylogenetic trees recovered different relationships between insular and continental *Eptesicus fuscus*, -ln -1899.58 and 18249.08 respectively. Bayesian analysis recovered a similar mitochondrial topology to plot (*a*), -ln -1932.27. Filled circles in (*a*,*b*) represent bootstrap support values of 100; white circles in (*a*) represent values between 96 and 99 and in (*b*) represent bootstrap values between 92 and 99. Shaded groups in (*a*) represent the species tree results from mPTP. (*c*) Population assignment (structure) DAPC analysis between Caribbean and continental lineages. (*d*) SVDQuartets analyses recovered a tree indicating a distinction between insular and continental *E. fuscus* with high support. Shaded groups in (*d*) represent species level differentiation.

Bayesian and maximum likelihood (ML) phylogenetic analysis of mitochondrial ND2 data recovered the Caribbean group defined herein as paraphyletic (figure 1), while supporting monophyly for a clade containing Mesoamerican and southern United States individuals. Besides Caribbean group paraphyly, trees showed each island as a separate monophyletic clade, and these are sister to southeastern United States individuals. To examine whether the clades recovered with mitochondrial ND2 data represent independent species, we used the tree-based coalescent species delimitation method mPTP. The three independent starting tree delimitation methods used in mPTP (null model, maximum likelihood or random) strongly inferred that each clade in the phylogeny represents a species (figure 1*a*).

We were interested in determining if the relationships observed using RADseq data supported the Caribbean group as a different species. The nuclear and mitochondrial tree topologies differed, with the RADseq maximum likelihood best-scoring tree suggesting the Caribbean group as monophyletic with respect to mainland taxa (figure 1*b*). Evaluation of genetic structure among geographic groups using DAPC showed that the Caribbean clade is highly structured and with no detectable admixture from individuals of other geographic groups. In contrast, mainland individuals from Mesoamerica and southeastern United States showed varying degrees of admixture (figure 1*c*). The DAPC plot, the minimum spanning network, and the genetic distance dendrogram with the supporting values can be found electronic supplementary material, figure S2.

The SplitsTree analysis indicated a lack of reticulated networks between the Caribbean lineages and continental relatives, suggesting low admixture of nuclear DNA for the insular group. This pattern stands in contrast to that seen in the mainland group which showed the presence of reticulate networks, suggesting past events of hybridization and recombination among the mainland lineages [49] (electronic supplementary material, figure S3). The Caribbean group had a higher average phylogenetic distance among individuals (µ 0.56) compared to distances among mainland individuals (µ 0.39). The phylogenetic distance between insular and mainland groups was still higher (µ 0.7), which supports the hypothesis of the Caribbean group as unique. Results from the species delimitation analysis based on SVDQuartets showed a tree topology supporting the hypothesis that the Caribbean clade should be considered a species. This relationship was recovered with high bootstrap support (figure 1*d*).

### 3.2. Assessment of phenotypic variation

**Figure 2.**
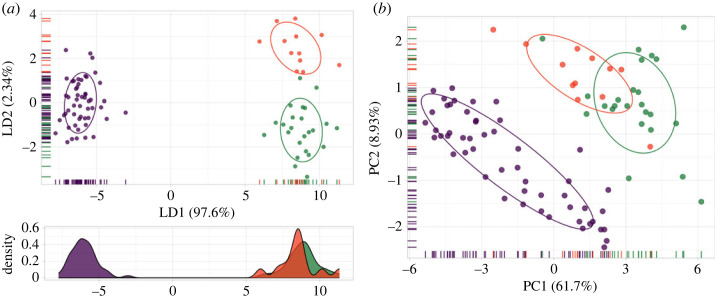
(*a*) Results from the machine learning linear discriminant analysis (LDA) of phenotypic limits for Caribbean *Eptesicus fuscus* lineages compared to mainland ones. The overall model accuracy was 98.86% (95% CI: 0.938, 0.9997). Solid lines represent 68% data ellipses cantered at the bivariate mean to visualize phenotypic differences among groups. Density values plotted to aid in visualization on the *x*-axis also show group distinction. (*b*) Principal component analysis (PCA) of phenotypic limits of the Caribbean *E. fuscus* lineages. Solid lines represent 68% of data ellipses to help visualize the discrimination between groups. The Caribbean group (in purple) was discriminated compared to the overlapping continental forms, i.e. southeastern United States (orange) and Mesoamerica (green).

The PCA testing for sexual dimorphism reflected that males and females included in the analysis broadly overlap across all 16 morphological characters used (electronic supplementary material, figure S4). LDA examining whether each group is phenotypically distinct achieved a discrimination proportion of 0.976 on LD1 and 0.023 on LD2 (figure 2). The machine learning LDA classifier of geographical partitions of *E. fuscus* had an accuracy of 98.86% (95% CI: 0.938, 0.9997) and was significantly better than the no-information rate (0.5909, *p* < 0.005). The LDA correctly classified the Caribbean and Mesoamerican morphotypes with 100% certainty. In contrast, one of the twelve specimens from southeastern United States was incorrectly classified as part of the Mesoamerican group (i.e. approx. 8% error).

The Caribbean group was clearly discriminated in the overall morphological space along the LD1. The two continental groups showed phenotypic overlap with no clear discrimination along LD1. The craniodental characters discriminating the Caribbean group from the others are associated with the length of the maxillary tooth row, the dentary length, and the rostral length (figure 2; electronic supplementary material, table S4 and figure S5). All geographical partitions showed a greater phenotypic overlap along the LD2, although this axis only explained about 2.3% of the overall group separation.

The PCA results strongly support the results of the LDA classifier (figure 2). The proportion of explained variance was 61.7% (95% CI: 61.58, 61.80) for PC1 and 8.93% (95% CI: 8.85, 9.01) for PC2. The Caribbean group was discriminated in morphological space, and the mainland groups showed overlap. Like LDA, the resulting groups from PCA primarily differed in characters associated with the length of the maxillary tooth row, the dentary length, the greatest skull length and the postorbital width.

### 3.3. Distribution and ecological niche differentiation

**Figure 3.**
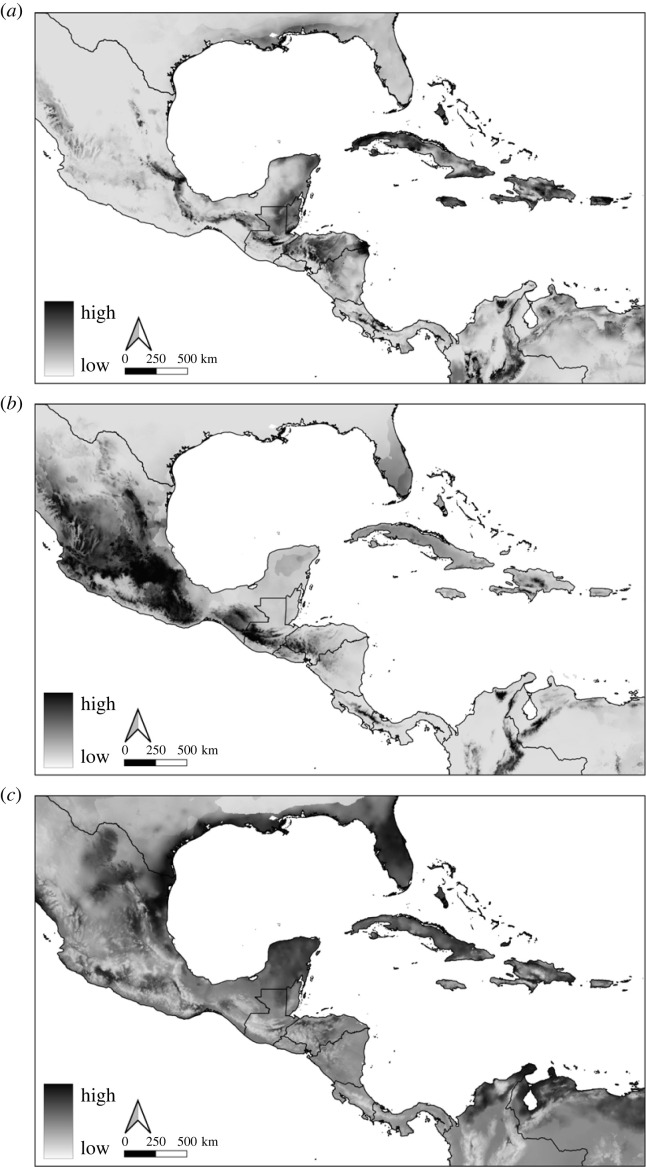
(*a*) Caribbean, (*b*) Mesoamerican, and (*c*) southeastern United States ecological niche model projections for *Eptesicus fuscus*. Noticeable differences in suitable habitats can be observed between the insular (*a*) and continental (*b*,*c*) *E. fuscus* groups. Models were calibrated with current location records and developed using Maxent v3.4.4. Darker regions represent habitats with higher suitability.

We generated ENMs from 404 georeferenced records arranged in three geographical partitions (i.e. Caribbean, Mesoamerica, and southeastern United States; electronic supplementary material, figure S1). The ENMs had good predictive performance (table 1) and showed evidence for environmental niche differentiation among the three geographic partitions of *E. fuscus* (figure 3). While false positives were present, all models showed higher suitability values in the specific geographical regions where the model was calibrated versus outside the calibration area. Estimated values of Schoener's *D* among pairwise comparisons for each group were characteristically low (table 2), indicating limited niche overlap between groups [79]. All tests of niche equivalency were statistically significant (*p* < 0.05) and supported our prediction that the compared niches between each group were not identical (electronic supplementary material, figure S6). Tests of niche identity showed that the estimated niches among groups are significantly different from each other (*p* < 0.05) [78,80]. Finally, the niche similarity tests (i.e. background similarity) revealed that the environments in which the partitions are structured do not significantly differ among groups.

**Table 2.** Pairwise comparison among three geographical partitions of *Eptesicus fuscus*. Estimations of niche overlap were based on statistical comparisons of niche equivalency (identity test) and background similarity (similarity test). The niche overlap statistic (Schoener's *D*) ranges from 0 (i.e. niches have no overlap) to 1 (i.e. niches are identical). Statistics with *p* < 0.05 represent ecological niches that are significantly different (i.e. not equivalent).

pairwise comparison	niche overlap (Schoener's *D*)	niche equivalency	niche similarity (symmetric test)
Caribbean versus Mesoamerica	*D* = 0.216	*p* < 0.05	*p* = 0.2
Caribbean versus southeastern US	*D* = 0.053	*p* < 0.05	*p* = 0.2
Mesoamerica versus southeastern US	*D* = 0.187	*p* < 0.05	*p* = 0.12

## 4. Discussion

Islands support a significant proportion of Earth's terrestrial species despite representing a small percentage of total land area on our planet [81]. Variations in degree of geographical isolation, past species interactions, and landscape variation are well-known factors that may drive the divergence between biological lineages [82]. These are expected to act as drivers of diversification in insular groups, triggering phylogenetic structural arrangements, morphological trait divergence and habitat shifts [83]. Ocean straits can act as barriers during diversification in insular bats despite their ability to fly; taxa that may be capable of flying between islands simply may not do so [26,84–86]. Isolation and lack of gene flow between island groups can result in well-defined bat clades associated with individual islands despite lack of other geographical constraints [87–89]. Similar geographically associated clades have been observed in insular birds [90]. Despite evidence of signals of deep divergence, the Caribbean and continental *E. fuscus* clades were still considered the same species [14,35,36,38] (but see [17]).

We revisited and expanded the analyses performed by Turmelle *et al*. [38] and Yi & Latch [14] to assess whether phylogenies for mitochondrial and nuclear data would provide evidence for Caribbean monophyly. The SNP data analyses presented in [14] did indicate divergence of *E. fuscus* in the Caribbean, and they highlighted the uniqueness of it as one of ‘five phylogeographical clades' forming ‘distinct conservation units’ (pp. 10–11). Similarly, Yi & Latch [36] using ultra-conserved element data considered individuals from the Caribbean to represent subspecies of *E. fuscus*, even though the clade was highly supported and showed an estimated divergence of 11 Ma from its sister mainland congeners (see figs 3 and 4 in [36]). These studies did not provide a basis for the species level recognition of Caribbean *E. fuscus*, despite comments alluding to this by Ramírez-Chaves *et al*. [17]. Herein, we re-evaluated these genetic data under a phylogenetic, population genomic and species delimitation framework to determine if Caribbean *E. fuscus* should be considered a separate species. Our nuclear phylogenetic analyses provided high support for monophyly of the Caribbean group, although analyses of mitochondrial data resulted in a different topology (figure 1*a*,*b*). The incongruence between mitochondrial and nuclear trees herein reflects the differential mode of inheritance of these genomes and could be a product of poor taxon sampling [91]. Notably, several insular groups were absent from the ND2 mitochondrial dataset (see [38]) and classical studies have shown that better taxon sampling can reduce bias and improve phylogenetic estimates [92]. Despite the topological incongruence between these datasets, there is still an underlying pattern of differentiation in the insular versus mainland groups that supports our hypothesis that Caribbean *E. fuscus* deserve species level recognition (figure 1). We elaborate this further.

Species delimitation analyses provided strong support for considering the Caribbean *E. fuscus* clade as a distinct species from mainland forms. First, the single locus mPTP analysis on the mitochondrial data recovered each clade as a species with strong support (figure 1*a*). We interpret the separation observed between individuals from Dominican Republic and Puerto Rico as evidence that additional diversity at the species level could be present. To further document the unique diversity of the Caribbean group at a genomic scale, we took a population genetic followed by a phylogenetic approach. We conducted DAPC analyses to evaluate the genetic structure among the biologically relevant data partitions. Our findings showed that the Caribbean group was highly structured and had no observed admixture with other partitions (figure 1*c*). The SplitsTree analysis showed the Caribbean group on a long branch that lacked reticulation, in contrast with the mainland groups that were connected in a web of reticulated networks (electronic supplementary material, figure S3). These findings provide evidence of likely reproductive isolation between continental and insular groups and of possible hybridization among continental lineages. Finally, the species delimitation analysis in SVDQuartets also strongly supported the hypothesis of the Caribbean *E. fuscus* as a species (figure 1*d*).

As a complement to the molecular analyses, we explored the phenotypic variation of three biologically relevant data partitions of *E. fuscus*: the Caribbean, Mesoamerica, and southeastern United States groups. Comparisons between mainland and insular groups detected a signal of divergence between the Caribbean and mainland in the form of distinctive craniodental relationships. Specifically, the Caribbean group was discriminated in morphological space with no overlap, in contrast to the overlapping mainland Mesoamerican and southeastern United States forms (figure 2*a*,*b*). The morphological disparity revealed by linear measurements showed proportional differences in length of different characters (electronic supplementary material, figure S5). Currently, the Caribbean *E. fuscus* subspecies are defined by morphological differences corresponding to geographical island limits [93]. We analysed a phenotypic dataset of five insular subspecies and found significant morphological variation among subspecies, a pattern that could explain the higher variation in morphological traits within the Caribbean clade compared to mainland forms (electronic supplementary material, figure S5 and table S4). We interpret the misidentification of some continental specimens (approx. 8% error rate) as evidence of morphological overlap between the two analysed continental phenotypes. Thus, obscuring the recognition of *E. f. miradorensis* as a species [17]. The examination of craniodental data supports the phylogenetic evidence presented that the Caribbean *E. fuscus* clade is a distinct species with a characteristic phenotypic pattern in the insular forms.

We hypothesized that environmental factors could reflect the differences observed in *E. fuscus* and would be congruent with a signal of speciation. Our niche modelling analysis indicated that the Caribbean and mainland groups occupy different niches with little to almost no overlap (figure 3; table 2). This supports the hypothesis that increased isolation and environmental distinctiveness of the Greater Antilles and The Bahamas could help maintain species level differences [82,94]. The environmental analyses of habitat suitability in geographic space for Caribbean *E. fuscus* revealed dissimilarities with potentially occupied habitats on the mainland (figure 3; table 2). The ecological niche differences between the Caribbean and the mainland *E. fuscus* lineages may be due to the availability of comparatively unfamiliar and unexploited terrain on the multiple colonized islands. Given the time of divergence of insular *E. fuscus* (*ca* 11 Ma [36]), it is probable that colonization of multiple Caribbean islands was facilitated by the narrowing of ocean straits during low sea stands in the Late Pleistocene. Our results rejected the niche equivalency hypothesis, suggesting that the small environmental similarities cannot explain the narrow but existent niche overlap (*D* = 0.216; table 2). Species limits of other widely distributed bat groups showed dynamic occupancy of insular niches compared to mainland congeners [5]. Additional data would be needed to quantify niche occupancy and examine the importance of Caribbean insular environments in maintaining species level differences in this system. Taken together, the disparities in which the Caribbean *E. fuscus* uses its available niche space also support the distinctiveness of this clade.

## 5. Conclusion

### 5.1. Species taxonomy and implications for the Caribbean

In this study, we described the signals of divergence supporting the Caribbean clade of *Eptesicus fuscus* as unique through an integrative framework. Our findings showed strong concordance among multiple methods, demonstrating that Caribbean *E. fuscus* have diagnostic features that fulfilled the species criteria of phylogenetic monophyly and craniodental distinctiveness, but also showed divergent ecological niche occupancy. Based on this integrative approach, we endorse the taxonomic recognition of Caribbean subspecies of *E. fuscus* at the species level. Following the principle of priority of the International Code of Zoological Nomenclature (ICZN, Article 23), the available name combination for this Caribbean taxon is *Eptesicus dutertreus* Gervais, 1837 (see *V*[*espertilio*] *dutertreus*, synonymy op. cit.). The holotype for this taxon is an adult male specimen collected in ‘Cuba’ by Ramón de la Sagra. No specific locality information provided. This specimen is preserved as a skull and skin (in alcohol) deposited at the Muséum national d'Histoire naturelle, Paris (France) with the specimen number MNHN-ZM-MO-1997-1832. This taxonomic arrangement includes all Bahamian and Greater Antillean subspecies (table 3). While *E. fuscus petersoni* was not included in our analyses due lack of access to cranial material, we highlight that measurements of the holotype of this subspecies match the lower end of the ranges for Caribbean *Eptesicus*. Specifically, the greatest skull length in the holotype of *E. f. petersoni* is 17.4 mm and the postorbital width is 3.7 mm [95]; our measurements show ranges of 17.5–20.2 mm and 3.61–4.53 mm, respectively.

**Table 3.** Revised taxonomy for subspecies of *Eptesicus dutertreus* and notes on their geographic distribution. Arranged alphabetically by subspecies.

species	subspecies	distribution
*Eptesicus dutertreus*	*bahamensis* Miller, 1897	The Bahamas: Abaco, Acklins, Andros, Crooked, Great Exuma, Little Exuma, Long, New Providence, and San Salvador. Type locality ‘Nassau, New Providence, Bahamas’
	*dutertreus* Gervais, 1837	Cuba. Type locality ‘Cuba’
	*hispaniolae* Miller, 1918	Hispaniola: Dominican Republic and Haiti. Type locality ‘Constanza, Santo Domingo’
	*lynni* Shamel, 1945	Jamaica
	*petersoni* Silva Taboada, 1974	Cuba: restricted to Isla de la Juventud. Type locality ‘Cueva de los Lagos, Cerro de las Guanábanas, Isla de Pinos’
	*wetmorei* Jackson, 1916	Puerto Rico. Type locality ‘Maricao, Puerto Rico’

Islands are considered hotspots of biodiversity often because of their high numbers of endemic species [96]. The insular Caribbean region is also infamous for its high rates of extinction for terrestrial mammals [97–100]. Species loss is exacerbated by the rapid pace of natural and human-driven habitat fragmentation and loss, and the low proportion of protected areas in many Caribbean islands [101]. The threats to West Indian bats that are considered Critically Endangered, Endangered, or Vulnerable all relate to human-driven factors [102]. Identifying the factors that threaten species is important to preserve biodiversity, but properly documenting potentially threatened taxa is key for successful conservation. Typically, the ecology and conservation status of widely distributed bats is evaluated based on generalized accounts of mainland populations and ignoring the smaller island populations despite their evolutionary potential, isolation, and unique threats. As currently assessed, *Eptesicus fuscus* *sensu lato*, is classified as Least Concern by the Red List of the International Union for the Conservation of Nature [103]. This includes both insular and mainland groups despite that island populations are rarely studied. Recognizing the Caribbean group as *Eptesicus dutertreus* can provide new grounds for the re-evaluation of the conservation status of this insular bat, identify its population trends, ecological preferences, and the factors that may threaten it on different islands to develop proper conservation plans for this species in the Caribbean.

## Data Availability

Results from additional analyses and datasets supporting this article have been uploaded as part of the electronic supplementary material. Genetic and genomic sequences were obtained from Genbank [38] and Dryad [14] (https://doi.org/10.5061/dryad.xsj3tx9h3) repositories. Text files of genetic, morphological, and spatial data, and R scripts used in this publication are deposited and freely accessible at https://doi.org/10.5281/zenodo.10412951 [104]. Supplementary material is available online [105].
